# Asymmetric kinase dimer formation is crucial for the activation of oncogenic EGFRvIII but not for ERBB3 phosphorylation

**DOI:** 10.1186/1478-811X-11-39

**Published:** 2013-06-10

**Authors:** Rama Krishna Kancha, Nikolas von Bubnoff, Justus Duyster

**Affiliations:** 1 Department Medicine I, University Medical Center Freiburg, Freiburg, Germany

**Keywords:** EGFRvIII, Asymmetric dimer, ERBB3, ERBB2, Lateral signaling

## Abstract

**Background:**

Formation of asymmetric kinase dimers is required for wt-EGFR activation upon ligand stimulation. The role of receptor dimerization in oncogenic EGFRvIII mutant activation is not completely understood and the molecular details of EGFRvIII interactions within homo-dimers and hetero-dimers are not elucidated yet.

**Findings:**

By employing mutations that disrupt the asymmetric kinase dimer interface in EGFRvIII, we demonstrate that the mechanism of oncogenic EGFRvIII mutant activation is similar to that of the full-length wild-type EGFR. Surprisingly, the monomeric EGFRvIII lacks autophosphorylation and the formation of asymmetric kinase dimers is indispensable for oncogenic kinase activation. In addition, we show that ERBB3 can act as an activator of EGFRvIII by forming asymmetric kinase dimer in a ligand-independent manner. Interestingly, we found that the formation of asymmetric kinase dimer is dispensable for ERBB3 phosphorylation by the activated EGFR kinase as well as the ERBB2 kinase thus revealing a novel model for receptor function.

**Conclusions:**

Lateral signaling is a novel mechanism of signal propagation via ERBB3 upon activation by EGFR/ERBB2 kinase even in the absence of their ability to form asymmetric kinase dimers.

## Findings

### EGFRvIII monomers lack kinase activity

**Figure 1:**
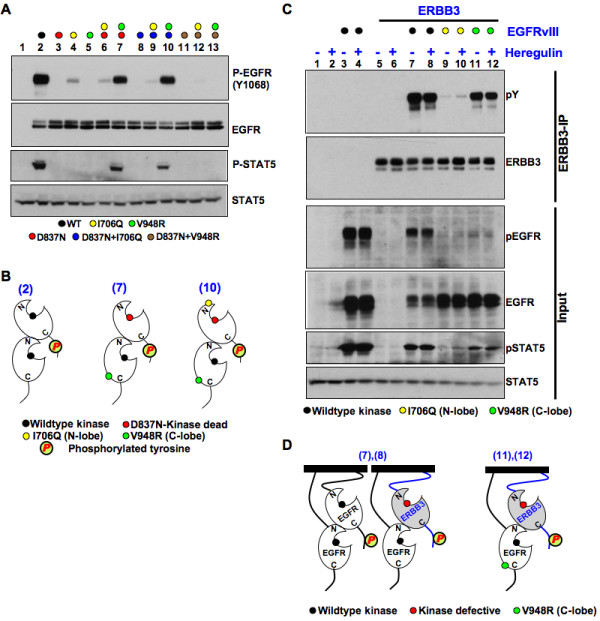
**Asymmetric kinase dimer formation is involved in EGFRvIII activation and ERBB3 phosphorylation. (A)** EGFRvIII point mutations were transfected into HEK293 cells either alone or in combinations as indicated by colored circles (lanes 2 to 13). Untransfected cells were taken as negative control (lane 1). Cell lysis was performed 36 hours after transfection followed by SDS-PAGE. Immunoblotting was performed with anti-p-EGFR (Y1068) (upper panel), anti-EGFR (second panel), anti-p-STAT5 (third panel) and anti-STAT5 (lower panel) antibodies. **(B)** Schematic representation of the most important mechanisms of wild-type and mutant EGFRvIII kinase activation corresponding to the experiments shown in Figure 1A in lanes 2, 7 and 10. Kinases were labeled black (intact active centre) or red (kinase dead) and yellow (N-lobe mutant) or green (C-lobe mutant) circles. **(C)** Wild-type or mutated EGFRvIII kinase (I706Q or V948R) was co-transfected with wild-type ERBB3 in HEK293 cells. After 36 hours, serum starvation for 12 hours was performed. Cells then were stimulated or not with 50 ng/ml heregulin for 5 minutes before cell lysis was performed. Immunoprecipitates with anti ERBB3 (upper 2 panels) or whole cell lysates (lower four panels) were resolved by SDS-PAGE and western blotting was performed with the antibodies indicated. **(D)** Schematic representation of key kinase domain interactions in receptor homo- and hetero-dimers corresponding to the experiments shown in Figure 1C in lanes 7, 8, 11 and 12.

Previous studies reported that EGFRvIII dimers cannot be detected in cells suggesting that kinase-active monomers of EGFRvIII execute mitogenic signal transduction [1-4]. This hypothesis is in accordance with the fact that EGFRvIII lacks the ligand-binding domain, which is critical for receptor dimerization. On the other hand subsequent reports were able to detect EGFRvIII dimers and could demonstrate that their activity is comparable to that of the ligand-stimulated wild-type EGFR [5,6]. Thus, the role of EGFRvIII dimerization for kinase activation is controversial and not fully elucidated. Recently, Zhang et al. using an elegant approach were able to demonstrate that the formation of asymmetric dimers of the kinase domain are absolutely essential for wt-EGFR activation upon ligand stimulation [7]. To test if asymmetric dimer formation is important for kinase activity we introduced point mutations (Additional file 1) in either the N-lobe or C-lobe of EGFRvIII and EGFRvIII-D837N (kinase-dead) (Figure 1A-B, Additional file 2: Figure S1A). We used cells that lack endogenous EGFR expression [8,9] (Figure 1A, lane 1). Disruption of the asymmetric kinase dimer interface by either N-lobe I706Q mutation (Figure 1A, lane 4) or C-lobe V948R mutation (Figure 1A, lane 5) indeed abrogated EGFRvIII kinase activity compared to the unmutated control (Figure 1A, lane 2). This data indicate that an intact asymmetric kinase dimer interface is essential for EGFRvIII kinase activation and was an unexpected finding given the previous observation that the receptor is not able to efficiently form stable dimers (Figure 1B, Additional file 2: Figure S1A) [1].

To further demonstrate the importance of asymmetric dimer formation for kinase activity, cells were transfected with a combination of mutants wherein the kinase activity of a C-lobe mutant (V948R) is rescued in *trans* by the kinase-dead (D837N) EGFRvIII either alone (Figure 1A-B, lane 7) or in combination with the N-lobe mutant (Figure 1A-B, lane 10). In contrast, the activity of C-lobe (V948R) mutant could not be rescued by a kinase-dead C-lobe mutant (D837N + V948R) due to the disruption of the asymmetric kinase dimer interface (Figure 1A-B, lane 13). Additional EGFRvIII mutants with disrupted asymmetric kinase dimer interface both in wild-type and D837N background were taken as controls (lanes 6, 8, 9, 11 and 12) to demonstrate the absence of *cis*-autophosphorylation (Figure 1A-B, Additional file 2: Figure S1A). Together these data argue for an important role of asymmetric dimer formation also for EGFRvIII kinase activation. Furthermore, it shows for the first time that the extra-cellular in-frame deletion of the EGFRvIII receptor does not result in an activated monomer as previously anticipated. A recent study reported the importance of Cys307 (wild-type EGFR numbering; Cys16 in mature EGFRvIII mutant) in EGFRvIII receptor dimerization [10]. We thus cloned the C16S mutant into the EGFRvIII/D837N backbone and tested for its ability to activate the C-lobe mutated EGFRvIII. As expected, the EGFRvIII/C16S + D837N mutant was not able to activate the EGFRvIII/V948R mutant indicating that the receptor dimerization is indispensable for EGFRvIII activity (Additional file 2: Figure S1B).

### ERBB3 is an activator of EGFRvIII in an asymmetric kinase hetero-dimer

Recently, it was shown that ERBB3 could act as an activator for the wt-EGFR kinase [11]. However, it is not known whether oncogenic EGFRvIII is able to form activating dimers with ERBB3. To test for potential ERBB3/EGFRvIII interactions, we expressed both constructs in HEK293 cells, which lack ERBB receptor expression [8,12] (Figure 1C: lanes 1–2, Additional file 3: Figure S2A). ERBB3 lacks intrinsic kinase activity [11] and when expressed alone didn’t cause receptor phosphorylation even in the presence of it’s ligand heregulin (Figure 1C: lanes 5–6, Additional file 3: Figure S2A). However, expression of ERBB3 together with EGFRvIII mutant resulted in ERBB3 phosporylation indicating that ERBB3 can act as a substrate for EGFRvIII kinase by forming heterodimers (Figure 1C-D: lanes 7–8). Interestingly, EGFRvIII-I706Q (N-lobe) mutant that disrupts asymmetric kinase dimer formation didn’t result in ERBB3 phosphorylation indicating that ERBB3 acts as an activator of EGFRvIII kinase (Figure 1C: lanes 9–10, Additional file 3: Figure S2A). Moreover, ERBB3 rescued the kinase activity of EGFRvIII-V948R (C-lobe) mutant thus demonstrating that the asymmetric kinase dimer interface is similar for both the EGFRvIII homodimers and EGFRvIII-ERBB3 heterodimers (Figure 1C-D: lanes 11–12). Receptor phosphorylation also correlated with the phosphorylation of downstream targets such as STAT5 indicating that the asymmetric kinase dimers are indeed functional. The phenomenon of EGFR kinase activation by asymmetric kinase dimerization thus seems to be highly conserved among different members of the EGFR family and different types of activating mutations found in human cancers [7,13]. Since kinase-inactive EGFRvIII is still able to be an activator for a partner receptor, inhibitor-bound-EGFRvIII may still activate other receptors of the EGFR family (ERBB2 or ERBB4). In the setting of EGFR tyrosine kinase inhibitor (TKI) treatment this may lead to altered signal transduction and secondary drug resistance. Thus, the expression of ERBB2, ERBB3 and ERBB4 in EGFR driven cancer may be important to predict the outcome of TKI treatment.

### Asymmetric kinase dimerization is dispensible for ERBB3 phosphorylation by activated EGFR and ERBB2 kinases

**Figure 2:**
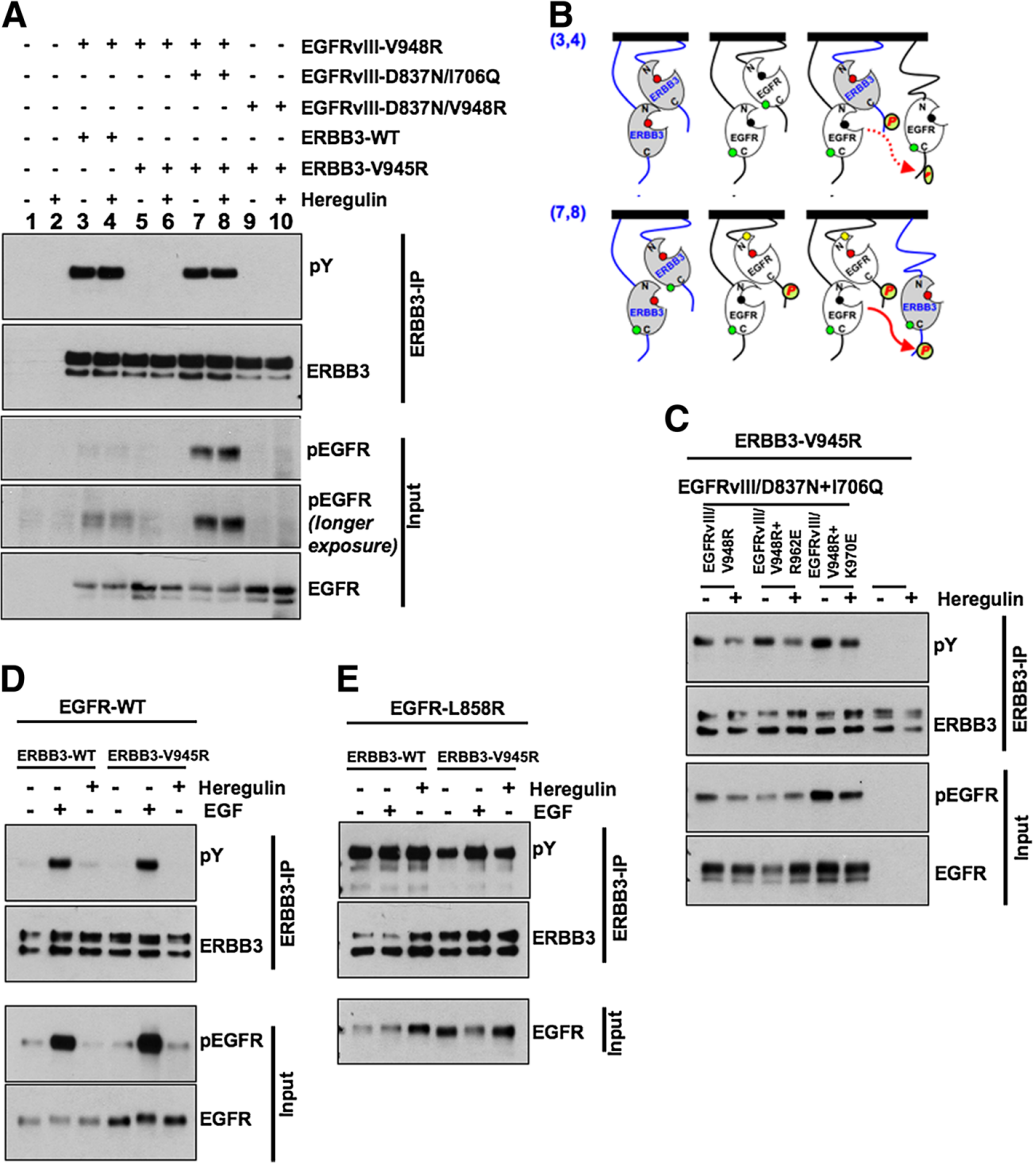
**Asymmetric kinase dimer formation is dispensable for ERBB3 activation by EGFR kinase. (A)** Indicated EGFRvIII and ERBB3 constructs were transfected into HEK293 cells. After 36 hours, stimulation with 50 ng/ml of heregulin for 5 minutes was performed. Cells were then lysed and subjected to immunoprecipitation using anti-ERBB3 antibody. Immunoblotting was performed on ERBB3 immunoprecipitates with indicated antibodies (top 2 panels). Whole cell lysates (input) were subjected to western blotting with indicated antibodies (lower 3 panels). **(B)** Schematic representation of receptor interactions corresponding to the experiments shown in Figure 2A (lanes 3, 4, 7 and 8). **(C)** Indicated EGFR and ERBB3 mutants were transfected into HEK293 cells followed by serum starvation and heregulin (50 ng/ml) stimulation for 5 minutes. Immunoblotting on ERBB3 immunoprecipitates was performed with indicated antibodies (upper 2 panels). Immunoblotting of whole cell lysates (input) was performed with indicated antibodies (lower 2 panels). **(D)** Wild-type EGFR was co-transfected with either wild-type or C-lobe mutant (V945R) ERBB3 into HEK293 cells. After 36 hours, cells were serum starved for 12 hours followed by stimulation with EGF (25 ng/ml) or heregulin (50 ng/ml) for 5 minutes. Cells were then lysed and subjected to ERBB3 immunoprecipitation. SDS-PAGE and immunoblotting on ERBB3 immunoprecipitates was performed with indicated antibodies (upper 2 panels). Western blotting on whole cell lysates (input) was performed with indicated antibodies (lower 2 panels). **(E)** EGFR-L858R was co-transfected with either wild-type or C-lobe mutant (V945R) ERBB3 into HEK293 cells. After 36 hours, cells were serum starved for 12 hours followed by stimulation with EGF (25 ng/ml) or heregulin (50 ng/ml) for 5 minutes. Cells were then lysed and subjected to ERBB3 immunoprecipitation. SDS-PAGE and immunoblotting on ERBB3 immunoprecipitates was performed with indicated antibodies (upper 2 panels). Western blotting on whole cell lysates (input) was performed with anti-EGFR antibody.

The dynamic role of monomers within an asymmetric dimer is not completely understood. It has been postulated that reciprocal asymmetric dimer formation, i.e. the acceptor kinase becomes the activator and vice versa leads to the full activation of both monomers [14]. It was also hypothesized that the activation by asymmetric kinase dimerization may happen in higher-order oligomers [14]. To address this we employed the EGFRvIII-ERBB3 heterodimer as a model wherein kinase activation can be viewed separately from substrate phosphorylation due to the differences in both size and the epitope. A series of EGFRvIII and ERBB3 mutants were created for this purpose. Consistent with the data shown in Figure 1C, EGFRvIII was able to phosphorylate ERBB3 in a ligand independent manner (Figure 2A-B: lanes 3, 4). ERBB3-V945R was previously shown to disrupt asymmetric kinase dimer formation [11] and as expected it failed to activate C-lobe (acceptor) mutant EGFRvIII (Figure 2A: lanes 5, 6, Additional file 3: Figure S2B). Surprisingly, when expressed together with an “activated EGFRvIII receptor homodimer unit”, ERBB3-V945R was phosphorylated (Figure 2A-B: lanes 7,8). This phenomenon was not observed when the “kinase-defective EGFRvIII activation unit” was used (Figure 2A: lanes 9,10, Additional file 3: Figure S2B) indicating that EGFRvIII is indeed the kinase. This finding is unexpected since ERBB3-V945R is defective in forming asymmetric kinase dimer with either of the EGFRvIII monomers used (EGFRvIII-D837N/I706Q and EGFRvIII-V948R). Thus, the lack of ability to act as an activator-kinase did not disqualify C-lobe (V945R) mutant ERBB3 to act as a substrate for EGFRvIII kinase. Symmetric kinase dimers were reported in EGFR crystals even though they have no role in kinase activation [7]. To test their role we introduced mutations (R962E and K970E) that disrupt the symmetric kinase interface of the activated (acceptor) kinase and expressed it together with ERBB3. Disruption of symmetric kinase dimer interface didn’t abrogate the ability of activated EGFRvIII kinase to phosphorylate ERBB3 (Figure 2C). Interestingly, a slight decrease in ERBB3 phosphorylation was observed upon heregulin stimulation (Figure 2C). It was previously shown that heregulin stimulation results in the destabilization of ERBB3 oligomers but not of receptor dimers [15]. Thus, the observed reduction of ERBB3 phosphorylation upon heregulin stimulation in this particular setting might be due to the disruption of higher-order oligmers involving ERBB3 receptors.

**Figure 3:**
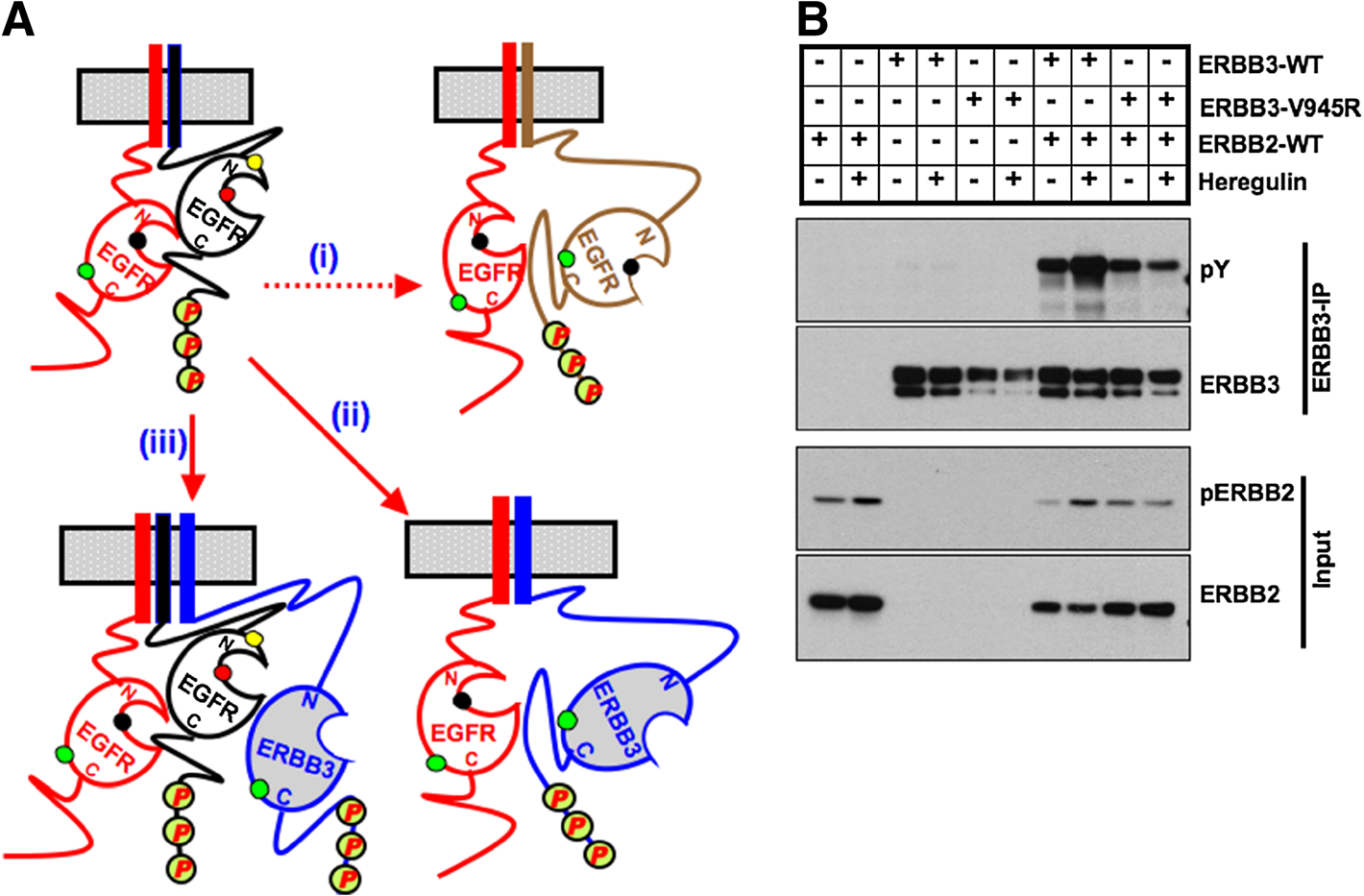
**Activated EGFR/ERBB2 kinase phosphorylates ERBB3 independent of asymmetric kinase dimer formation. (A)** Schematic representation of two hypothetic mechanisms potentially involved in activator function-defective EGFR/ERBB3 phosphorylation by activated EGFR: The activated EGFR dissociates from the activator kinase to phosphorylate another inactive (activator function-defective) EGFR (i) or ERBB3 (ii) receptor. Alternatively, activated EGFR may form higher-order oligomer or hetero-tetramer complexes to cause phosphorylation of activator function-defective ERBB receptors (iii). **(B)** HEK293 cells were transfected with wild-type ERBB2 either alone or together with WT or mutant ERBB3 (V945R). After 36 hours, cells were serum starved for 12 hours followed by stimulation with 50 ng/ml heregulin for 5 minutes as indicated. Cells were then lysed in TMNSV buffer [16]. Cells lysates were subjected to immunoprecipitation using rabbit anti-ERBB3 antibody and immunoblotting was performed using mouse anti-phospho-tyrosine and mouse anti-ERBB3 antibodies (upper 2 panels). Western blotting on whole cell lysates (input) was performed with anti-pERBB2 and anti-ERBB2 antibodies (lower 2 panels).

To test if similar mechanisms are involved in ERBB3 phosphorylation by the full-length receptor, we expressed both wild-type and C-lobe mutant ERBB3 together with the wild-type EGFR. Interestingly, full length, wild-type EGFR phosphorylated both wild-type as well as the C-lobe mutant ERBB3 upon EGF stimulation but not upon heregulin stimulation (Figure 2D). EGFR-L858R, a constitutively active mutant that was reported in NSCLC patients, also phosphorylated both wt and C-lobe mutant ERBB3 in a ligand-independent manner (Figure 2E). These results imply that it is not necessary for ERBB3 to be part of an asymmetric kinase dimer to be activated by EGFR kinase. The phosphorylation of ERBB3 by EGFR kinase despite the inability to form neither asymmetric nor symmetric dimers of kinase domain indicates that the dimerization or oligomerization at receptor level brings the acceptor kinase close enough to phosphoryate it’s substrate (Figure 3A). Moreover, ERBB2 phosphorylated both the wild-type and C-lobe mutant ERBB3 (Figure 3B) indicating that the observed mechanism is conserved among different members of the ERBB family.

### Activated ERBB3 potentiates the transforming ability of EGFR and ERBB2

**Figure 4:**
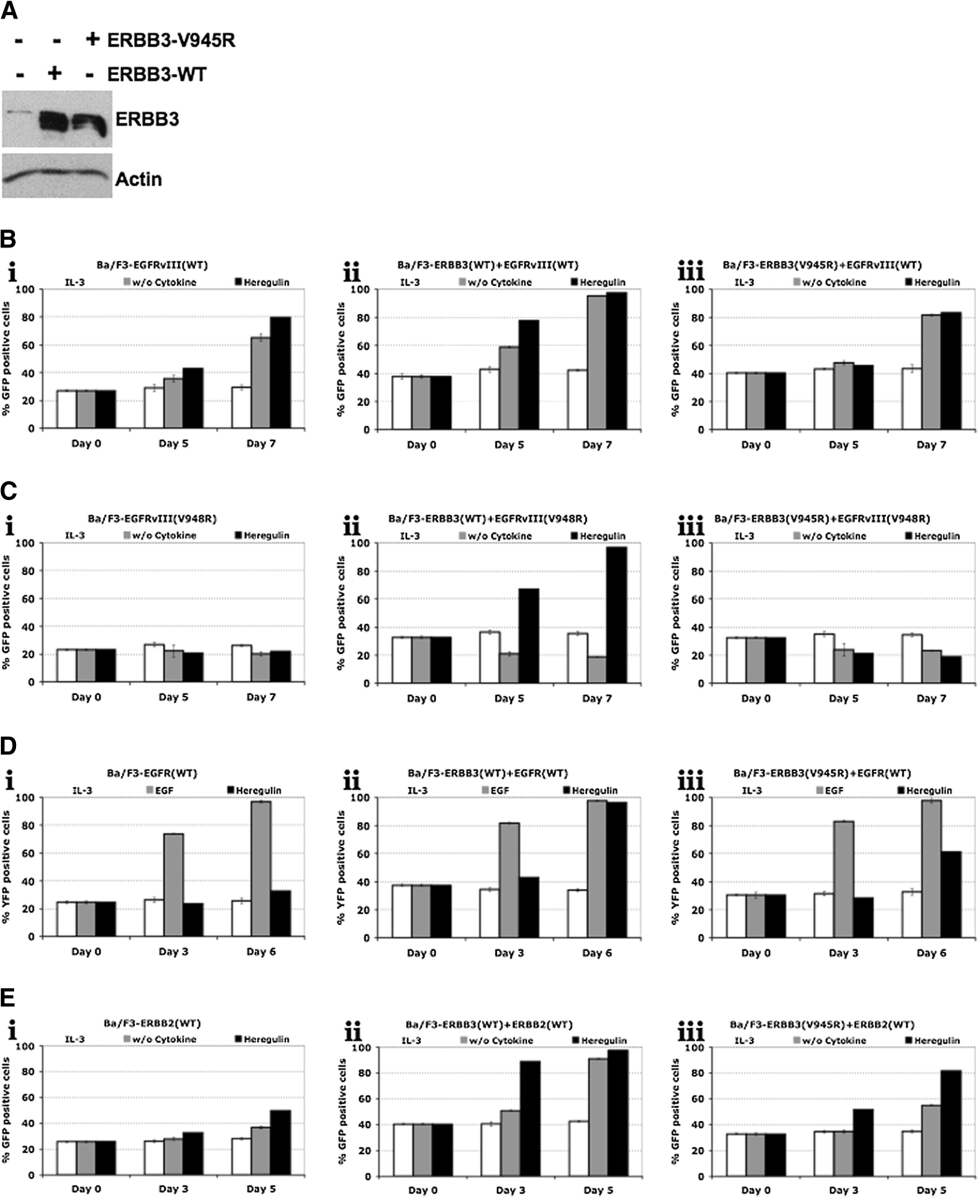
**Functional analysis of ERBB heterodimers. (A)** Ba/F3 cells stably expressing indicated ERBB3 receptors were lysed and western blotting was performed with anti-ERBB3 and anti-beta-actin antibodies. Untransfected Ba/F3 cells were taken as a negative control. **(B**-**E)** Ba/F3 cell lines described above with or without stable expression of ERBB3 constructs were additionally infected with MSCV-GFP-EGFRvIII (**B**: i: control, ii: ERBB3-WT, iii: ERBB3-V945R), MSCV-GFP-EGFRvIII/V948R (**C**: i: control, ii: ERBB3-WT, iii: ERBB3-V945R), MSCV-YFP-EGFR (**D**: i: control, ii: ERBB3-WT, iii: ERBB3-V945R) or MSCV-GFP-ERBB2 (**E**: i: control, ii: ERBB3-WT, iii: ERBB3-V945R). Cells were then cultured with or without the indicated cytokines (IL-3, EGF (25 ng/ml) or heregulin (50 ng/ml). The Percentage of GFP-positive (EGFRvIII or ERBB2) or YFP (EGFR-WT)-positive cells was measured by FACS analysis at the indicated time points.

To test the functional role of kinase activation within different ERBB receptor combinations, we employed a competition assay using Ba/F3 cells. Ba/F3 cells lack ERBB receptor expression and were previously used to test the transformation potential of ERBB receptors [8,9,12]. Ba/F3 cells require IL-3 for survival and introduction of oncogenes into these cells confer cytokine-independence on these cells [8,9,12]. Thus, we employed Ba/F3 cells to test the physiological role of ERBB3 phosphorylation by ERBB receptors in different combinations. Stable cell lines expressing either wild-type or C-lobe mutant ERBB3 (V945R) were established for this purpose (Figure 4A). Both wild-type and C-lobe mutant ERBBs (EGFR, EGFRvIII and ERBB2) were transduced in to either parental Ba/F3 cells (Figure 4B-E: panel i) or Ba/F3 cells that stably express ERBB3 receptors (Figure 4B-E: panels ii and iii). Since EGFR, EGFRvIII and ERBB2 were expressed from bi-cistronic vectors that co-express either GFP or YFP, the signaling strength of receptor combinations was measured as the rate of outgrowth of transduced cells over untransduced cells [9,12]. EGFRvIII transformed Ba/F3 cells to cytokine-independence as reported previously (Figure 4B: i) [8,9]. The transformation of Ba/F3 cells by EGFRvIII is potentiated by the co-expression of ERBB3 receptor indicating an important role of ERBB3 phosphorylation (Figure 4B: ii, iii). As expected, the C-lobe mutant EGFRvIII-V948R didn’t transform Ba/F3 cells (Figure 4C: i). However, expression of ERBB3 receptor conferred IL3-independence on Ba/F3 cells that stably express EGFRvIII-V948R indicating the functional role of asymmetric kinase dimer formation (Figure 4C: ii). EGFRvIII-V948R however failed to transform Ba/F3 cells that expressed ERBB3-V945R even after heregulin stimulation indicating the importance of asymmetric kinase dimer interface in the activation of receptor (Figure 4C: iii). Moreover, transformation of Ba/F3 cells to IL-3 independence by full-length wild-type EGFR was also potentiated by ERBB3 upon heregulin stimulation (Figure 4D: i, ii). Even though similar patterns were seen with cells expressing ERBB3-V945R, the transformation ability was relatively weak compared to that of cells expressing wild-type ERBB3 (Figure 4D: ii, iii). Similar observations were made with the combination of ERBB2 and ERBB3 receptors (Figure 4E: i - iii) indicating that the signaling strength resulting from the lateral signaling is weaker compared to that of the ‘activating asymmetric kinase dimer’ unit.

## Conclusions

Formation of asymmetric kinase dimer is essential for both the constitutive activation of oncogenic EGFR as well as the ligand-stimulated wild-type EGFR. However, phosphorylation of ERBB3 by the activated EGFR or ERBB2 kinase may occur in higher order oligomers in the absence of asymmetric kinase dimer formation. Thus, asymmetric kinase dimer formation plays a differential role in EGFR receptor activation and ERBB3 phosphorylation. Recent studies have implicated the role of ERBB3 as a critical heterodimeric partner for both EGFR and ERBB2 in drug resistance [17]. Since the formation of receptor complexes is important for their activity, the use of antibodies that target ERBB receptors either alone or in combination with ERBB inhibitors might abrogate the development of secondary drug resistance.

## Competing interest

The authors have no competing interests to declare.

## Authors’ contributions

RKK: Conceived the study, designed and performed experiments, analyzed data and wrote the manuscript. NvB: Conceived the study and analyzed data. JD: Conceived the study, analyzed data and wrote the manuscript. All authors read and approved the final manuscript.

## Supplementary Material

Additional file 1 Methods description. Click here for file

Additional file 2: Figure S1 (A) Schematic representation of the mechanism of wild-type or mutant EGFRvIII interactions for corresponding lanes in Figure 1A (numbered 2 to 13). Kinases were labeled with black (intact active centre) or red (kinase dead) and yellow (N-lobe mutant) or green (C-lobe mutant) circles. Intact or rescued kinase activity is represented by a phospho-tyrosine (*P*). (B) Wild-type (WT) or mutated EGFRvIII (KD-Kinase defective) were transfected into HEK293 cells either alone or in combinations as indicated. Untransfected cells (UT) were taken as a negative control (lane 1). Cell lysis was performed 36 hours after transfection followed by SDS-PAGE. Immunoblotting was performed with anti-p-EGFR (Y1068) and anti-EGFR antibodies. Click here for file

Additional file 3: Figure S2 (A) Schematic representation of receptor interactions shown in Figure 1B. (B) Schematic representation of receptor interactions in the setting of both homo- and hetero-dimers. Numbers correspond to lanes in the Figure 2A. Click here for file
