# Supplementary material for: Asymmetric kinase dimer formation is crucial for the activation of oncogenic EGFRvIII but not for ERBB3 phosphorylation
Source: Cell Commun Signal. 2013 Jun 10;11:39. doi: 10.1186/1478-811X-11-39 (PMC3726407; doi:10.1186/1478-811X-11-39)
Supplement: Additional file 1 — Methods description. [file 1478-811X-11-39-S1.pdf]

Additional file 1

Methods

Reagents, DNA constructs and cell lines

Recombinant human EGF was purchased from Chemicon (Millipore). Recombinant heregulin was purchased from Calbiochem. MiGR1-EGFRvIII and pcDNA-ERBB3 were described previously[8, 9, 11]. Point mutations that disrupt the asymmetric kinase dimer interface were introduced into MiGR1-EGFRvIII as described previously[8, 9, 11]. HEK293 cells were cultured in DMEM (Life Technologies) supplemented with 10% FCS. Ba/F3 cells were cultured in RPMI 1640 (Life Technologies) supplemented with 10% FCS, glutamine and interleukin-3 (R&D Systems).

Immunoprecipitation and western blotting

HEK293 cells were transfected with indicated constructs using Lipofectamine 2000 reagent (Invitrogen) for 36 hours and cell lysis was performed as described previously[8]. HEK293 cells that were transfected with both EGFR/ERBB2 and ERBB3 were serum starved for 12 hours followed by stimulation with EGF or heregulin. ERBB3 immunoprecipitation was performed using rabbit anti-ERBB3 (Santa Cruz Biotechnology) antibody after lysing the cells with TMNSV buffer [15]. Immunoblotting was performed using following antibodies: p-EGFR (Y1068, Cell Signaling), EGFR (Santa Cruz Biotechnology), anti-pSTAT5-Y694 (Cell Signaling), anti-STAT5 (Santa Cruz Biotechnology), anti-phosphotyrosine-4G10 (Upstate Biotechnology), anti-phosphotyrosine-PY20 (BD Biosciences), and mouse anti-ERBB3 (Santa Cruz Biotechnology).

Competition assay

Ba/F3 cells were transduced with either wild-type (a kind gift from David Riese, Purdue University) or C-lobe mutant (V945R) ERBB3 using retroviral spin-infection method and stable cell lines were established by G418 selection for one week. 1 x 10^6 cells in 4 ml medium were then subjected to IL3-withdrawal or cultured in IL-3, EGF (25 ng/ml) or heregulin (50 ng/ml). Percentage of GFP-positive or YFP-positive cells was measured by FACS analysis at indicated time points.
