# Supplementary material for: Asymmetric kinase dimer formation is crucial for the activation of oncogenic EGFRvIII but not for ERBB3 phosphorylation
Source: Cell Commun Signal. 2013 Jun 10;11:39. doi: 10.1186/1478-811X-11-39 (PMC3726407; doi:10.1186/1478-811X-11-39)

# Supplementary figure 1

## A. Schematic representation of kinase interactions from Figure 1A

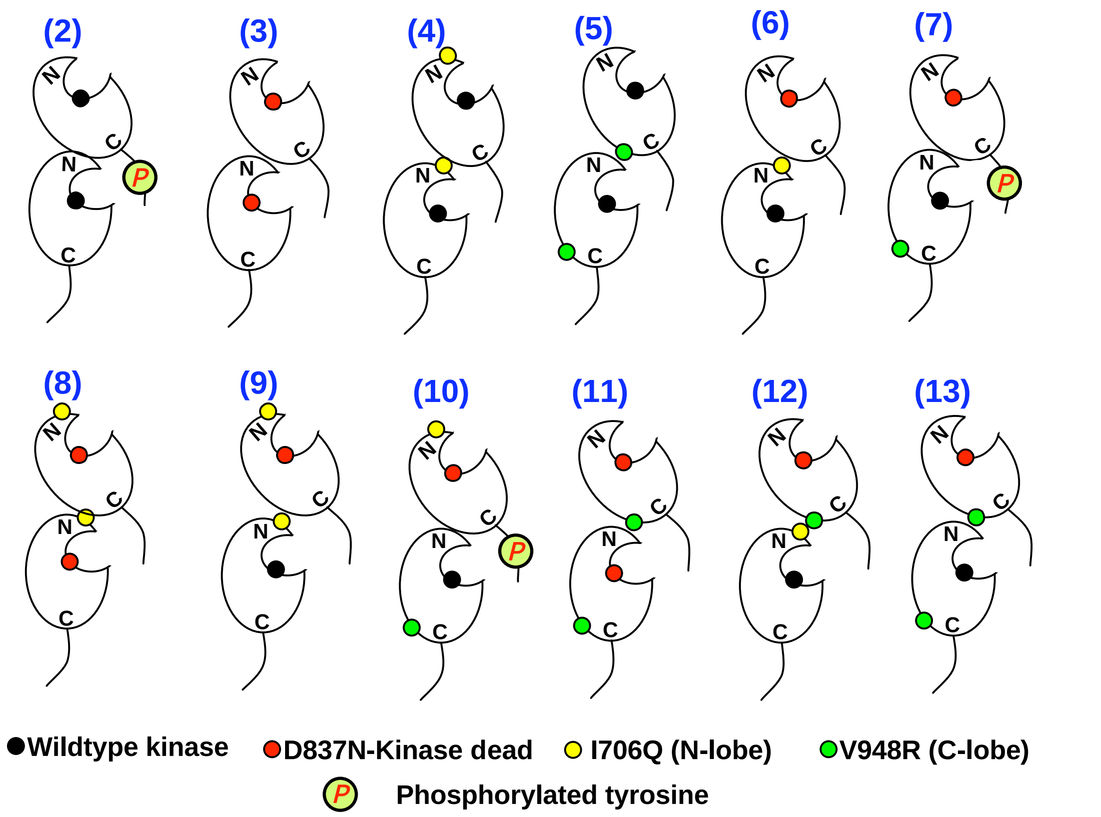


## B. HEK293 cells were transfected with indicated EGFRvIII mutants and their activity was analyzed by western blotting (UT-Untransfected control, WT-Wild-type EGFRvIII, KD-Kinase-dead EGFRvIII)

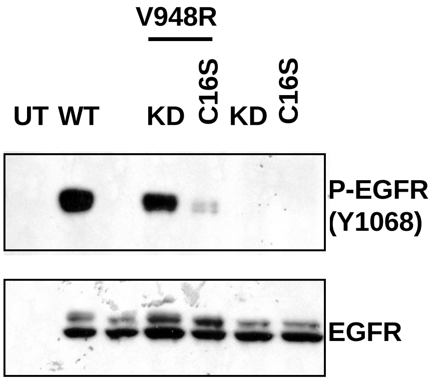

Supplement: Additional file 2: Figure S1 — (A) Schematic representation of the mechanism of wild-type or mutant EGFRvIII interactions for corresponding lanes in Figure 1A (numbered 2 to 13). Kinases were labeled with black (intact active centre) or red (kinase dead) and yellow (N-lobe mutant) or green (C-lobe mutant) circles. Intact or rescued kinase activity is represented by a phospho-tyrosine (P). (B) Wild-type (WT) or mutated EGFRvIII (KD-Kinase defective) were transfected into HEK293 cells either alone or in combinations as indicated. Untransfected cells (UT) were taken as a negative control (lane 1). Cell lysis was performed 36 hours after transfection followed by SDS-PAGE. Immunoblotting was performed with anti-p-EGFR (Y1068) and anti-EGFR antibodies. [file 1478-811X-11-39-S2.pdf]
