# Supplementary material for: Asymmetric kinase dimer formation is crucial for the activation of oncogenic EGFRvIII but not for ERBB3 phosphorylation
Source: Cell Commun Signal. 2013 Jun 10;11:39. doi: 10.1186/1478-811X-11-39 (PMC3726407; doi:10.1186/1478-811X-11-39)

Supplementary figure 2

A. Schematic representation of receptor interactions from the Figure 1B

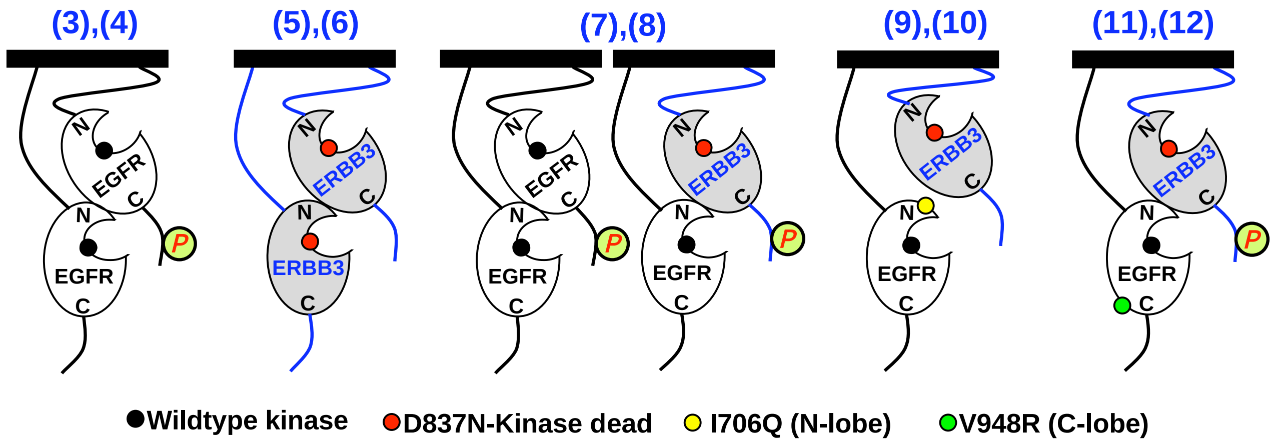

B. Schematic representation of receptor interactions from the Figure 2A

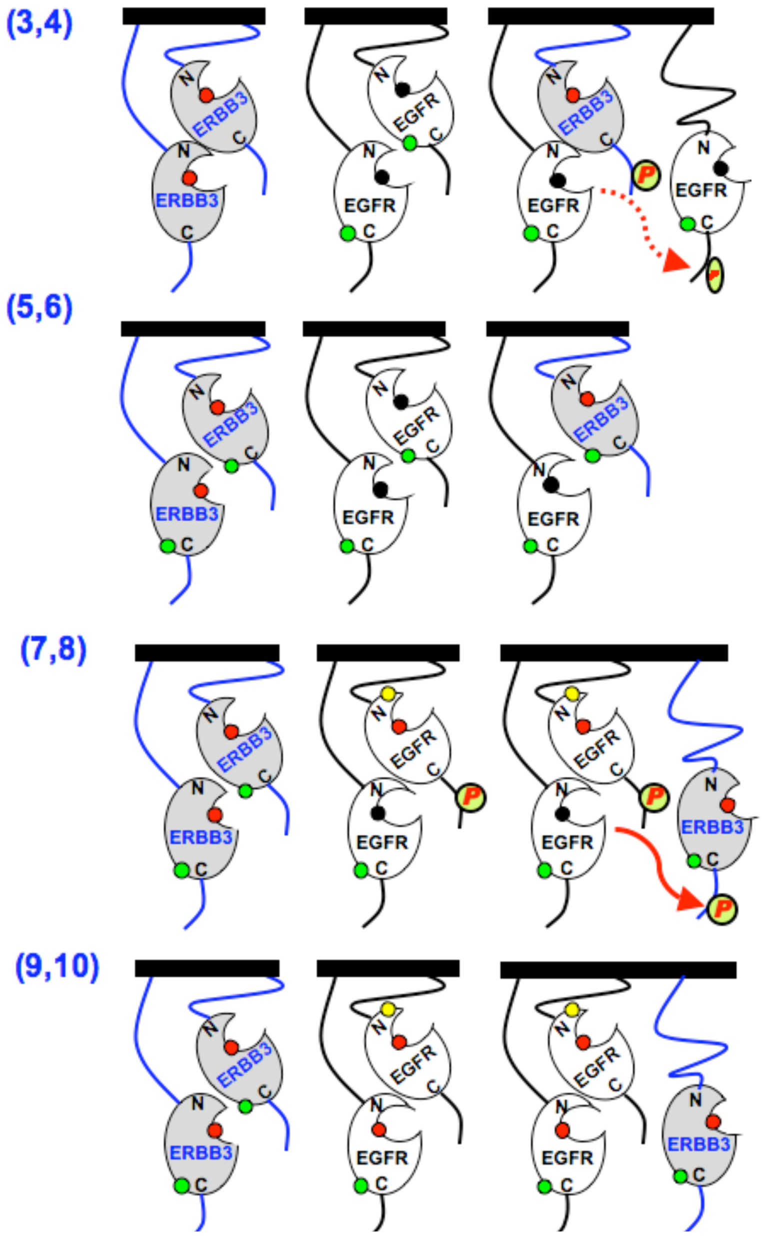

Supplement: Additional file 3: Figure S2 — (A) Schematic representation of receptor interactions shown in Figure 1B. (B) Schematic representation of receptor interactions in the setting of both homo- and hetero-dimers. Numbers correspond to lanes in the Figure 2A. [file 1478-811X-11-39-S3.pdf]
